# The Effect of Age and Recent Influenza Vaccination History on the Immunogenicity and Efficacy of 2009–10 Seasonal Trivalent Inactivated Influenza Vaccination in Children

**DOI:** 10.1371/journal.pone.0059077

**Published:** 2013-03-12

**Authors:** Sophia Ng, Dennis K. M. Ip, Vicky J. Fang, Kwok-Hung Chan, Susan S. Chiu, Gabriel M. Leung, J. S. Malik Peiris, Benjamin J. Cowling

**Affiliations:** 1 School of Public Health, Li Ka Shing Faculty of Medicine, The University of Hong Kong, Hong Kong Special Administrative Region, China; 2 Department of Microbiology, Li Ka Shing Faculty of Medicine, The University of Hong Kong, Hong Kong Special Administrative Region, China; 3 Department of Pediatrics and Adolescent Medicine, Li Ka Shing Faculty of Medicine, The University of Hong Kong, Hong Kong Special Administrative Region, China; 4 Centre for Influenza Research, Li Ka Shing Faculty of Medicine, The University of Hong Kong, Hong Kong Special Administrative Region, China; University of Melbourne, Australia

## Abstract

**Background:**

There is some evidence that annual vaccination of trivalent inactivated influenza vaccine (TIV) may lead to reduced vaccine immunogenicity but evidence is lacking on whether vaccine efficacy is affected by prior vaccination history. The efficacy of one dose of TIV in children 6–8 y of age against influenza B is uncertain. We examined whether immunogenicity and efficacy of influenza vaccination in school-age children varied by age and past vaccination history.

**Methods and Findings:**

We conducted a randomized controlled trial of 2009–10 TIV. Influenza vaccination history in the two preceding years was recorded. Immunogenicity was assessed by comparison of HI titers before and one month after receipt of TIV/placebo. Subjects were followed up for 11 months with symptom diaries, and respiratory specimens were collected during acute respiratory illnesses to permit confirmation of influenza virus infections. We found that previous vaccination was associated with reduced antibody responses to TIV against seasonal A(H1N1) and A(H3N2) particularly in children 9–17 y of age, but increased antibody responses to the same lineage of influenza B virus in children 6–8 y of age. Serological responses to the influenza A vaccine viruses were high regardless of vaccination history. One dose of TIV appeared to be efficacious against confirmed influenza B in children 6–8 y of age regardless of vaccination history.

**Conclusions:**

Prior vaccination was associated with lower antibody titer rises following vaccination against seasonal influenza A vaccine viruses, but higher responses to influenza B among individuals primed with viruses from the same lineage in preceding years. In a year in which influenza B virus predominated, no impact of prior vaccination history was observed on vaccine efficacy against influenza B. The strains that circulated in the year of study did not allow us to study the effect of prior vaccination on vaccine efficacy against influenza A.

## Introduction

Receipt of trivalent inactivated influenza vaccination (TIV) should stimulate a robust immune response including rises in humoral antibody titers against vaccine strains [1]. Rises in antibody titer occur 2–3 weeks after vaccination and persist for a few months [2]. The proportion of vaccine recipients achieving post-vaccination antibody titers ≥1∶40 by hemagglutination inhibition (HI) is of particular interest because this threshold has been correlated with 50% protection against infection in historical studies [3], [4], although those studies mostly did not involve children [4]. Vaccine efficacy is measured in randomized controlled trials as the degree of protection conferred against laboratory-confirmed influenza virus infection.

In some countries children are recommended to receive influenza vaccination every year, and a concern has been raised over whether repeated vaccination affects vaccine efficacy and long term immunity in children [5]. Previous studies examining antibody responses to repeated influenza vaccination have been inconclusive [6]–[19]. Few studies have investigated whether vaccine efficacy or effectiveness against laboratory-confirmed influenza virus infection in vaccinated individuals is affected by their vaccination history [20]–[23]. The current dosage recommendation for children 6 months to 8 years of age also considers vaccination history of these children [24]. Very few studies have however evaluated the effect of previous vaccination on vaccine efficacy or effectiveness, particularly in children between 6 years and 8 years of age [25], [26].

In a large trial we randomly allocated 796 school age children to receive seasonal TIV or placebo [27], [28]. In a separate study of 64 children who were also randomly allocated to receive TIV or placebo in the preceding year, we reported that receipt of TIV in the first year was associated with reduced antibody responses to TIV in the second year for the influenza A vaccine strains which were the same in both years [29]. As the majority of those 64 children were older than 8 years of age, we could not explore whether prior vaccination of the same vaccine virus also affected antibody responses in younger children. In this report, we analysed data from all 796 children included in the trial to explore whether the effect of prior vaccination on antibody response to TIV was age dependent and to what extent it might affect vaccine efficacy. We also examined the effect of previous vaccination on the efficacy of one dose of TIV in children aged 6 to 8 years of age and 9–17 years of age against influenza B which predominated during the study period.

## Methods

### Subjects and Follow Up

TIV and placebo were randomly allocated to study subjects from August 2009 through January 2010 [28]. Eligible subjects were between 6 and 17 years of age and not contraindicated for receipt of TIV. At the time of enrollment, the study subjects and their parents were interviewed by a trained research assistant to obtain information regarding their influenza vaccination status for the 2007–08 and 2008–09 influenza seasons (i.e. one and two years earlier), medical history and other personal and household demographics. Sixty-four of the children had also participated in the study in the preceding year [27], and information on receipt of TIV as part of that trial is included in this study.

Serum specimens were collected immediately before and 1 month after vaccination, during follow-up (April-May, 2010, “mid-study”) and at the end of the follow up period (August-December, 2010). All subjects and their household members were monitored for signs and symptoms of acute upper respiratory tract infections (URTIs) through biweekly telephone interviews and daily symptom diaries, and reminders to notify our study team about any illnesses. Following identification of an acute URTI in any study subject or household member, we arranged home visits to collect nose and throat swabs from all household members (regardless of illness). The case definition used to trigger a home visit was at least 2 of fever ≥37.8°C, cough, sore throat, headache, chills, coryza or myalgia. The study was approved by the Institutional Review Board of the University of Hong Kong. All participants 18 years or older gave written informed consent. Proxy written consent from parents or legal guardians was obtained for persons 17 years or younger, with additional written assent from those 8 to 17 years of age.

### Vaccines

Study subjects were randomized to receive one dose of TIV (0.5 ml VAXIGRIP, Sanofi Pasteur) or placebo (0.5 ml saline) in the ratio 3∶2. The seasonal TIV used in this study contained A/Brisbane/59/2007(H1N1)-like, A/Brisbane/10/2007(H3N2)-like and B/Brisbane/60/2008-like strains. The TIV and placebo were packaged in identical syringes by trained staff not involved in vaccine administration to preserve the double-blind study design. Children 6–8 years old who had never received influenza vaccination before were only given one dose of TIV, rather than the recommended 2 doses, based on the hypothesis that children in Hong Kong could be more experienced with influenza virus infection so that one dose may be sufficiently immunogenic [30]. The vaccine compositions recommended by the World Health Organization for the Northern Hemisphere in the previous years, and used in Hong Kong, were A/Solomon Islands/3/2006 (H1N1)-like, A/Wisconsin/67/2005 (H3N2)-like and B/Malaysia/2506/2004-like viruses (2007–08); and A/Brisbane/59/2007 (H1N1)-like, A/Brisbane/10/2007 (H3N2)-like and B/Florida/4/2006-like viruses (2008–09) (Table 1). Only inactivated influenza vaccines were licensed in Hong Kong at the time; live attenuated influenza vaccines were first registered in Hong Kong during our study period, and no subjects in our study reported receipt of live attenuated influenza vaccines. Monovalent inactivated pandemic influenza A(H1N1pdm09) vaccine became available during our study period but community uptake was low and none of the subjects in our study reported receipt of that vaccine.

**Table 1:** Antigenic relationship between vaccine virus strains included in the trivalent inactivated influenza vaccines for the 2007–2010 seasons (Northern Hemisphere).

Season	Seasonal influenza A(H1N1)	Seasonal influenza A(H3N2)	Seasonal influenza B
2007–2008 [41]	A/Solomon Islands/3/2006	A/Wisconsin/67/2005	B/Malaysia/2506/2004 (Victoria-lineage)
2008–2009 [42]	A/Brisbane/59/2007 (Cross reactivewith post A/SolomonIslands/3/2006 infection ferretsera [43])	A/Brisbane/10/2007 (Highly crossreactive with postA/Wisconsin/67/2005infection ferret sera [43])	B/Florida/4/2006 (Yamagata-lineage)
2009–2010 [44]	Same as previous year	Same as previous year	B/Brisbane/60/2008 (Victoria-lineage)

### Laboratory Methods

Serum specimens were tested by HI assays with serial dilutions from 1∶10 to 1∶1280, to estimate antibody titers against the vaccine strains A/Brisbane/59/2007(H1N1), A/Brisbane/10/2007-like virus A/Uruguay/716/2009 (H3N2) and B/Brisbane/60/2008 (Victoria-lineage), and the prevalent pandemic influenza strain A/California/7/2009 (H1N1pdm09). Sera with antibody titers ≥1∶1280 were re-titrated to endpoint. Nose and throat swabs were tested by reverse transcription polymerase chain reaction (RT-PCR) for influenza A and B viruses. The HI assay and RT-PCR were performed using standard methods as reported previously [27], [31].

### Statistical Methods

Geometric mean antibody titers before and 1 month after receipt of TIV or placebo were compared between children who received influenza vaccination in any of the previous 2 years and those who did not, using Wald tests after log transformation of the antibody titers. The geometric mean titer ratio was compared using Wald tests after log transformation of the titer ratio one month after receipt of TIV to before receipt. Non-parametric comparisons using Wilcoxon signed- rank tests were also performed, using data without multiple imputations. The results were stratified to examine whether prior vaccination had differential effect on antibody response in younger (6–8 years old) and older children (9–17 years old). The proportion of children attaining antibody titer of ≥1∶40 and ≥1∶160 following vaccination were compared using combined chi-squared tests. Antibody titers <1∶10 were imputed as 1∶5. Multiple imputations for a small amount of missing data were performed using 10 imputations, to make use of all available data [32]. Multiple imputations were performed using an algorithm involving predictive mean matching [33]–[35].

We used multivariable Poisson regression models allowing for the duration of follow-up in each subject to model how the risk of RT-PCR-confirmed influenza virus infections was affected by age, presence of chronic health conditions, receipt of TIV/placebo and prior vaccination history. Analyses were stratified by age, and an interaction term between TIV/placebo and prior vaccination was included in the models. Vaccine efficacy was estimated as (1-incidence rate ratio)x100%. All analyses were performed using R version 2.10.1 (R Foundation for Statistical Computing, Vienna, Austria).

## Results

Of 796 study subjects, 479 were randomized to TIV and 317 to placebo. One subject allocated to placebo withdrew from the study after randomization but before the intervention was administered. Of the 479 children who received TIV, 150 (31%) of them had received TIV in either 2007–08 or 2008–09, and 51 (11%) children suffered from chronic health conditions such as allergic rhinitis or asthma (Table 2). Children allocated to placebo had similar characteristics (data not shown) [31]. The timeline and the weekly number of influenza virus infections confirmed by RT-PCR during the study are shown in Figure S1. Children were followed up for a median of 351 days (interquartile range 327–371 days) following receipt of TIV/placebo, including periods when influenza B viruses from the Victoria lineage predominated. The activities of circulating influenza strains detected by RT-PCR in our study closely matched with influenza circulation in Hong Kong based on data from the reference laboratory for Hong Kong Island at Queen Mary Hospital [31].

**Table 2:** Characteristics of 479 subjects randomized to receive trivalent inactivated influenza vaccine (TIV) in 2009–10 with regard to their vaccination history in 2007–08 and 2008–09.

	Not received TIVin 2007–08or 2008–09		Received TIV in2007–08 only		Received TIV in2008–09 only		Received TIV in2007–08& 2008–09		Incomplete vaccination history	
	n	(%)	n	(%)	n	(%)	n	(%)	n	(%)
**6–8 years of age**	94		15		40		17		4	(2)
Male	44	(47)	12	(80)	20	(50)	8	(47)		
Presence of chronic health conditions	7	(7)	1	(7)	4	(10)	1	(6)		
**9–17 years of age**	221		22		41		15		10	(3)
Male	110	(50)	14	(64)	22	(54)	11	(73)		
Presence of chronic health conditions	28	(13)	5	(23)	3	(7)	2	(13)		

**Figure 1:**
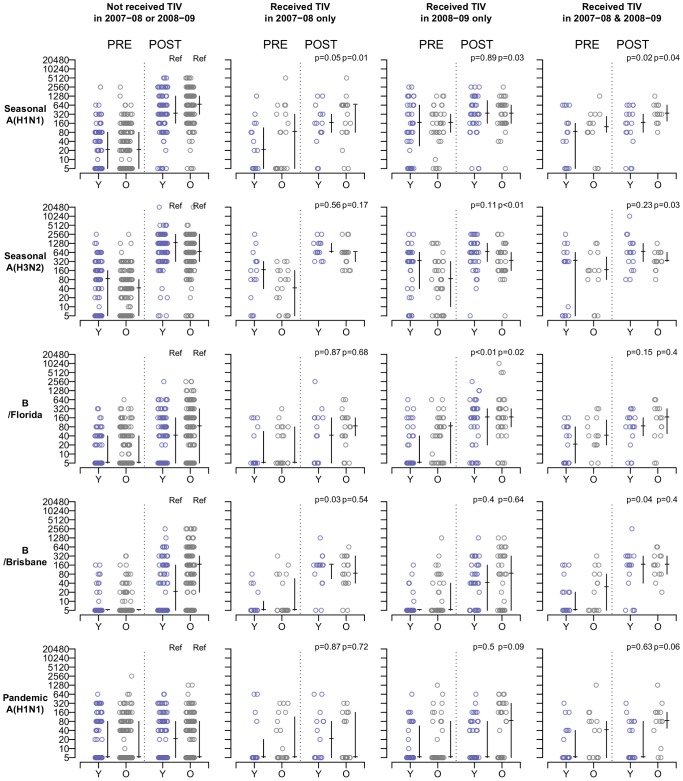
Individual antibody titers before and one month after receipt of trivalent inactivated influenza vaccine (TIV) in 2009–2010 among 6–8 y (Y, represented by blue circles) and 9–17 y children (O, represented by grey circles) with regard to their vaccination history for the 2007–2008 and 2008–2009 seasons. The median and interquartile range of antibody titers are shown, p-values were obtained by non parametric Wilcoxon signed rank tests. The comparisons were made with reference to children who were randomized to receive TIV in 2009–10 but did not receive any TIV during 2007–2008 and 2008–2009 seasons. The two p-values shown in each plot were obtained by comparison with children of the same age in the corresponding reference group (6–8 y and 9–17 y).

To explore the potential influence of age and prior vaccination history on antibody response to vaccine, antibody titers before and one month after receipt of 2009–10 TIV were studied among younger (6–8 y) and older (9–17 y) school age children with different vaccination history. The vaccine virus strains included during the previous seasons (Northern Hemisphere) are shown in Table 1. Individual antibody titers are shown in Figure 1 and the corresponding summary statistics are shown in Table S1 and Table S2. A general pattern was observed particularly among older children on the effect of previous vaccination on antibody response to seasonal influenza A(H1N1) and A(H3N2) vaccine viruses. Previously vaccinated children tended to show higher pre-vaccination antibody titer but lower post-vaccination antibody titer to the seasonal A vaccine strains. Nevertheless, they still had high post-vaccination antibody titers.

A different effect of previous vaccination was observed on the antibody response to seasonal influenza B vaccine virus particularly among younger children. There were a considerable number of younger children who failed to mount a high antibody response to the 2009–10 study TIV if they had not had previously received the 2007–08 vaccine which included a Victoria-lineage influenza B virus. Receipt of the 2007–08 vaccine was associated with an improved antibody response to 2009–10 vaccine virus of the same lineage. However, similar enhancement effect was not observed if the previous influenza B vaccine virus belonged to the Yamagata-lineage.

The pandemic influenza A(H1N1) virus was not included in any of the 2007–08, 2008–09 or 2009–10 vaccines. Neither the 2007–08 or 2008–09 TIV appeared to affect antibody response to the 2009–10 TIV against pandemic influenza A(H1N1). However, it appears that older children who had received TIV for 3 consecutive years showed small increase in antibody titer to pandemic influenza A(H1N1) virus. It is uncertain whether that might have been affected by recent infections with the circulating pandemic influenza A(H1N1) virus.

For comparison we investigated the subjects who received placebo in 2009–10 and we found that previous vaccination did not affect antibody response to placebo and the antibody profile was consistent with vaccination history (Figure S2, Table S3 and Table S4).

To investigate whether previous vaccination affected vaccine efficacy, the incidence of RT-PCR confirmed influenza infection among the children was examined. Influenza B virus (Victoria-lineage) dominated during the study and detection rate of influenza A viruses was low (Figure S1). Table 3 shows TIV had 60–70% efficacy against confirmed influenza B virus infection in all age groups regardless of vaccination history. There was no evidence that previous vaccination negatively affected vaccine efficacy. The results also show 1 dose of TIV was of comparable efficacy in preventing seasonal influenza B among 6–8 y Hong Kong children even if they had not been vaccinated in recent years. Further study is however required to confirm the efficacy against influenza A.

**Table 3:** Factors affecting risk of RT-PCR confirmed influenza B infection and vaccine efficacy (VE) of trivalent inactivated influenza vaccine (TIV) in 2009–10.

		n		RR	(95% CI)		P-value		VE		(95% CI)		
	**6–8 years of age**												
Model 1	Placebo	104	1.00										
	TIV, not vaccinated in 2007–08	136	0.23		(0.06,	0.83)	0.02	77%		(17%,		84%)	
	TIV, vaccinated in 2007–08	32	0.30		(0.04,	2.39)	0.26	70%		(−139%,		96%)	
Model 2	Placebo	104	1.00										
	TIV, not vaccinated in 2008–09	109	0.38		(0.12,	1.21)	0.10	62%		(−21%,		88%)	
	TIV, vaccinated in 2008–09	57	–					–					
Model 3	Placebo	104	1.00										
	TIV, not vaccinated in 2007–08 or 2008–09	98	0.33		(0.09,	1.20)	0.09	67%		(−20%,		67%)	
	TIV, vaccinated in 2007–08 or 2008–09	72	0.14		(0.02,	1.06)	0.06	86%		(−6%,		98%)	
	**9–17 years of age**												
Model 1	Placebo	213		1.00									
	TIV, not vaccinated in 2007–08	262		0.33	(0.12,	0.93)	0.04	67%		(7%,		88%)	
	TIV, vaccinated in 2007–08	37		0.45	(0.06,	3.52)	0.45	55%		(−252%,		94%)	
Model 2	Placebo	213		1.00									
	TIV, not vaccinated in 2008–09	245		0.36	(0.13,	1.02)	0.05	64%		(−2%,		87%)	
	TIV, vaccinated in 2008–09	61		0.29	(0.04,	2.20)	0.23	71%		(−120%,		96%)	
Model 3	Placebo	213		1.00									
	TIV, not vaccinated in 2007–08 or 2008–09	226		0.32	(0.10,	0.98)	0.05	68%		(2%,		68%)	
	TIV, vaccinated in 2007–08 or 2008–09	83		0.42	(0.25,	1.89)	0.26	58%		(−89%,		75%)	

Footnote: Relative risks (RR) were approximated by the incidence rate ratio, adjusted for presence of chronic health conditions. “–” represents statistic that could not be estimated due to lack of RT-PCR confirmed influenza B infection in this category.

## Discussion

While past vaccination history did affect the antibody responses to TIV, we found that vaccine efficacy against confirmed influenza B did not appear to be affected by past vaccination history. Our estimates for vaccine efficacy were consistent with the values typically observed in other studies in the range 60%–70% [36]. There is little information in the literature on how previous influenza vaccination might affect vaccine efficacy. A number of studies have demonstrated that repeated annual vaccination can lead to poorer antibody responses [6]–[14], [20], although this is not always the case [8], [18], [19].

In our previous study, we reported reduced antibody response to seasonal influenza A(H1N1) and A(H3N2) vaccine virus in children aged 6–17 years if the vaccine viruses remained unchanged from the previous year [29]. In this report, we explored the role of antigenic similarity and age on the interaction of previous vaccination on antibody response to TIV. We found that previous exposure of the same seasonal influenza A(H1N1) and A(H3N2) vaccine virus reduced antibody response to TIV, and the effect appeared to be more profound in older children. The effect of previous exposure to antigenically related seasonal influenza A(H1N1) and A(H3N2) vaccine viruses included in the 2007–08 influenza vaccine was also found to negatively affect antibody response. However, it remains unclear whether this change in immunogenicity translates into any changes in vaccination efficacy due to low circulation of seasonal influenza A(H1N1) and A(H3N2) viruses during the study. Furthermore, most of the vaccinated children attained post-vaccination antibody titers of ≥1∶40 to seasonal influenza A(H1N1) and A(H3N2) viruses regardless of vaccination history.

It was observed in this study that 1 dose of TIV elicited lower antibody responses to influenza B among children aged 6–8 y if they had not been recently vaccinated although this did not appear to lead to lower efficacy. Previous vaccination was only observed to improve antibody response to influenza B vaccine virus if the current vaccine strain belonged to the same virus lineage. It is uncertain if the dosage recommendation for 6 m-9 y children should also consider the lineage of influenza B vaccine virus they had previously received. However this may not be feasible in practice, and a preferable solution could be the introduction of quadrivalent influenza vaccines covering both Victoria and Yamagata lineages. It remains to be determined whether antibody response measured by the HI assay is a good predictor of protection and whether current threshold for vaccine licensure is appropriate in children. There remain uncertainties about the 50% protective antibody titer in children [37], [38]. In our study we observed that TIV remained efficacious even though some children showed low antibody titers after vaccination.

There are several limitations in our study. Firstly, previous receipt of influenza vaccine was within the discretion of the participants and their parents. Any systematic differences among previously vaccinated children might have confounded our results. It was reassuring that the antibody profile of children receiving placebo did not differ by vaccination history. The patterns in antibody response was also consistent with our previous study on the subset of 64 children who had also been randomly allocated to TIV/placebo in the preceding year [29]. However, ascertainment bias might still occur if previously vaccinated children were more likely to report their illness. On the other hand, it is also possible that these parents may adopt better household and personal hygiene measures so that previously vaccinated children may be less exposed to influenza viruses. As parents might have difficulty recalling earlier vaccination history, only vaccination history of 2007–08 and 2008–09 was obtained. Errors in self-reported vaccination history and smaller numbers of younger children may have also affected our power to detect differences in risk of confirmed infections. However, the antibody profile generally matched with the reported vaccination history in our study. One other limitation is that only a small proportion of children reported previous vaccination and this limited the power of our study to detect changes in vaccine efficacy in relation to vaccination history. It has been reported that HI may not detect protective antibody that do not bind to the haemagglutinin [39] and HI titer may not be strongly correlated with immunity to influenza. We did not measure neutralizing antibodies in our study, although there is limited information in the literature on how neutralizing antibodies may provide additional information on protection against infection compared with HI titers [40]. While our study did not find that previous vaccination affected vaccine efficacy against influenza B, further study is required to confirm whether repeated exposure of antigenically identical or related influenza A vaccine viruses may affect vaccine efficacy. It is also noted that the peri-pandemic period may not be the best time to study immunogenicity and efficacy of seasonal influenza vaccine although influenza B did predominate in our study period.

In conclusion, antibody responses in children to the strains included in the 2009–10 seasonal TIV were affected by age and previous vaccination history. Previously vaccinated children showed reduced antibody responses against seasonal A(H1N1) and seasonal A(H3N2) particularly in older children. Regardless of past influenza vaccination history, antibody titers to seasonal influenza A were raised to high levels following administration of TIV in children in our study. Previous vaccination was associated with higher antibody responses to influenza B in younger children although we did not find any effect on vaccine efficacy. The strains that circulated in the year of study did not allow us to study the effect of prior vaccination on vaccine efficacy against influenza A.

## Supporting Information

Figure S1 **Weekly number of RT-PCR confirmed influenza infections and the time-line of the vaccination trial.**
(TIF)

Figure S2 **Individual antibody titers before and one month after receipt of placebo in 2009–2010 among 6–8 y (Y, represented by blue circles) and 9–17 y children (O, represented by grey circles) with regard to their vaccination history for the 2007–2008 and 2008–2009 seasons.** The median and interquartile range of antibody titers are shown, p-values were obtained by non parametric Wilcoxon signed rank tests. The comparisons were made with reference to children who were randomized to receive TIV in 2009–10 but did not receive any TIV during 2007–2008 and 2008–2009 seasons. The two p-values shown in each plot were obtained by comparison with children of the same age in the corresponding reference group (6–8 y and 9–17 y).
(TIF)

Table S1 **Comparison of antibody titers before and 1 month after receipt of 2009–10 trivalent inactivated influenza vaccine (TIV) in children 6–8 years of age with regard to their vaccination history**
(DOCX)

Table S2 **Comparison of antibody titers before and 1 month after receipt of 2009–10 trivalent inactivated influenza vaccine (TIV) in children 9–17 years of age with regard to their vaccination history.**
(DOCX)

Table S3 **Comparison of antibody titers before and 1 month after receipt of placebo in children 6–8 years of age with regard to their vaccination history.**
(DOCX)

Table S4 **Comparison of antibody titers before and 1 month after receipt of placebo in children 9–17 years of age with regard to their vaccination history.**
(DOCX)
