# Supplementary figures and images for: The Effect of Age and Recent Influenza Vaccination History on the Immunogenicity and Efficacy of 2009–10 Seasonal Trivalent Inactivated Influenza Vaccination in Children
Source: PLoS One. 2013 Mar 12;8(3):e59077. doi: 10.1371/journal.pone.0059077 (PMC3595209; doi:10.1371/journal.pone.0059077)

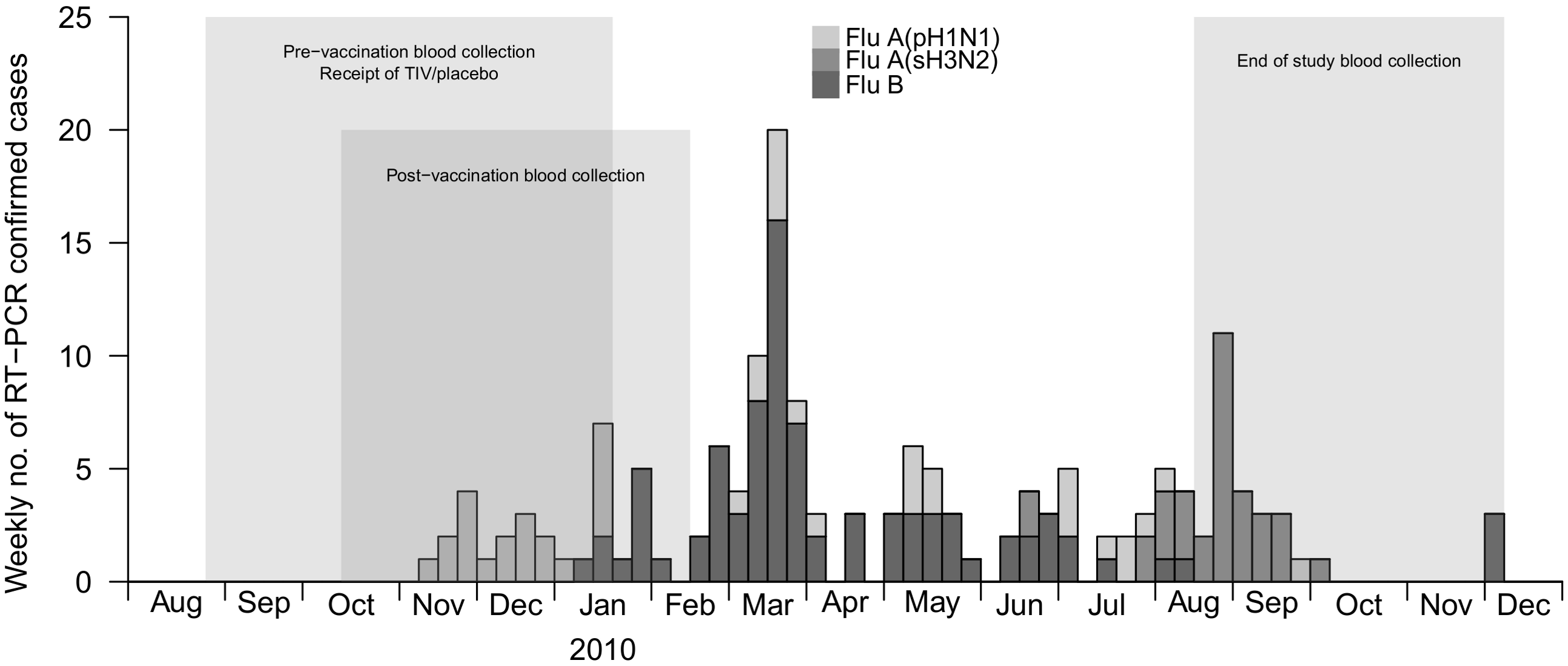

Supplement: Figure S1 — Weekly number of RT-PCR confirmed influenza infections and the time-line of the vaccination trial. (TIF) [file pone.0059077.s001.tif]

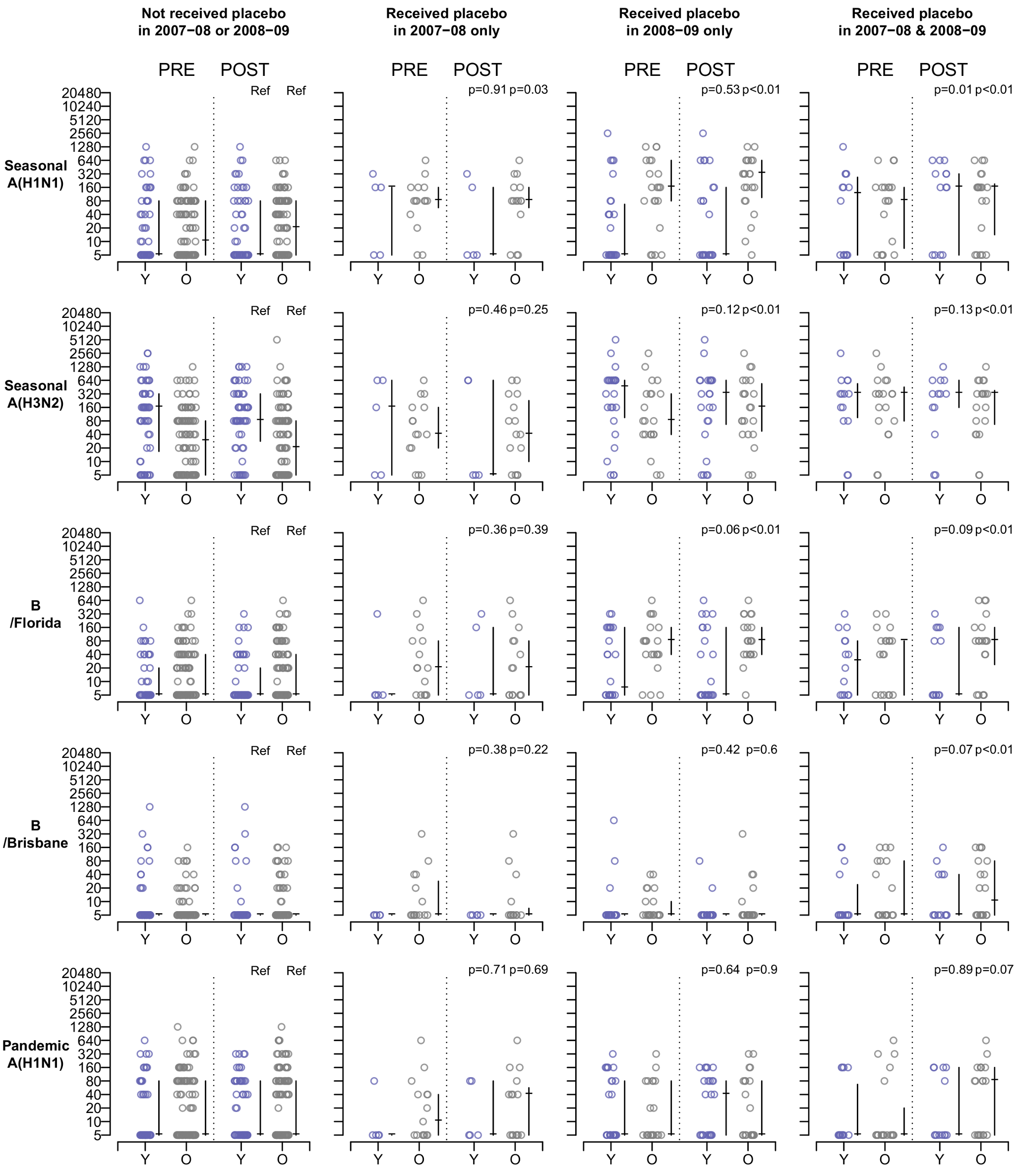

Supplement: Figure S2 — Individual antibody titers before and one month after receipt of placebo in 2009–2010 among 6–8 y (Y, represented by blue circles) and 9–17 y children (O, represented by grey circles) with regard to their vaccination history for the 2007–2008 and 2008–2009 seasons. The median and interquartile range of antibody titers are shown, p-values were obtained by non parametric Wilcoxon signed rank tests. The comparisons were made with reference to children who were randomized to receive TIV in 2009–10 but did not receive any TIV during 2007–2008 and 2008–2009 seasons. The two p-values shown in each plot were obtained by comparison with children of the same age in the corresponding reference group (6–8 y and 9–17 y). (TIF) [file pone.0059077.s002.tif]
