# Supplementary material for: The Effect of Age and Recent Influenza Vaccination History on the Immunogenicity and Efficacy of 2009–10 Seasonal Trivalent Inactivated Influenza Vaccination in Children
Source: PLoS One. 2013 Mar 12;8(3):e59077. doi: 10.1371/journal.pone.0059077 (PMC3595209; doi:10.1371/journal.pone.0059077)
Supplement: Table S1 — Comparison of antibody titers before and 1 month after receipt of 2009–10 trivalent inactivated influenza vaccine (TIV) in children 6–8 years of age with regard to their vaccination history (DOCX) [file pone.0059077.s003.docx]

Table S1. Comparison of antibody titers before and 1 month after receipt of 2009-10 trivalent inactivated influenza vaccine (TIV) in children 6-8 years of age with regard to their vaccination history.

|  | Reference |  | Comparison 1 | |  | Comparison 2 | |  | Comparison 3 | |
| --- | --- | --- | --- | --- | --- | --- | --- | --- | --- | --- |
|  | Not received TIV in 2007-08 or 2008-09 |  | Received TIV in 2007-08 only | P-value |  | Received TIV in 2008-09 only | P-value |  | Received TIV in 2007-08 & 2008-09 | P-value |
|  | (n=94) |  | (n=15) |  |  | (n=72) |  |  | (n=17) |  |
| Seasonal A(H1N1) |  |  |  |  |  |  |  |  |  |  |
| Before vaccination |  |  |  |  |  |  |  |  |  |  |
| GMT | 24 |  | 32 | 0.55 |  | 105 | <0.01 |  | 54 | 0.12 |
| proportion ≥1:40 | 0.45 |  | 0.48 | 0.79 |  | 0.75 | 0.01 |  | 0.65 | 0.49 |
| 1 month after vaccination |  |  |  |  |  |  |  |  |  |  |
| GMT | 320 |  | 139 | 0.08 |  | 314 | 0.95 |  | 115 | 0.03 |
| proportion ≥1:40 | 0.92 |  | 0.87 | 0.92 |  | 0.93 | 0.93 |  | 0.83 | 0.41 |
| GMTR | 14.0 |  | 4.0 | 0.03 |  | 3.0 | <0.01 |  | 2.0 | <0.01 |
|  |  |  |  |  |  |  |  |  |  |  |
| Seasonal A(H3N2) |  |  |  |  |  |  |  |  |  |  |
| Before vaccination |  |  |  |  |  |  |  |  |  |  |
| GMT | 45 |  | 108 | 0.11 |  | 160 | <0.01 |  | 112 | 0.13 |
| proportion ≥1:40 | 0.59 |  | 0.74 | 0.67 |  | 0.78 | 0.10 |  | 0.65 | 1.00 |
| 1 month after vaccination |  |  |  |  |  |  |  |  |  |  |
| GMT | 771 |  | 825 | 0.78 |  | 554 | 0.20 |  | 637 | 0.59 |
| proportion ≥1:40 | 0.95 |  | 1.00 | 0.94 |  | 0.97 | 1.00 |  | 1.00 | 0.86 |
| GMTR | 17.0 |  | 8.0 | 0.17 |  | 3.0 | <0.01 |  | 6.0 | 0.05 |
|  |  |  |  |  |  |  |  |  |  |  |
| Seasonal B/Brisbane |  |  |  |  |  |  |  |  |  |  |
| Before vaccination |  |  |  |  |  |  |  |  |  |  |
| GMT | 7 |  | 9 | 0.33 |  | 7 | 0.56 |  | 15 | 0.02 |
| proportion ≥1:40 | 0.09 |  | 0.20 | 0.63 |  | 0.13 | 1.00 |  | 0.23 | 0.29 |
| 1 month after vaccination |  |  |  |  |  |  |  |  |  |  |
| GMT | 31 |  | 103 | 0.01 |  | 41 | 0.42 |  | 93 | 0.03 |
| proportion ≥1:40 | 0.50 |  | 0.87 | 0.05 |  | 0.66 | 0.51 |  | 0.77 | 0.22 |
| GMTR | 5.0 |  | 12.0 | 0.05 |  | 6.0 | 0.58 |  | 6.0 | 0.54 |
|  |  |  |  |  |  |  |  |  |  |  |
| Pandemic A(H1N1) |  |  |  |  |  |  |  |  |  |  |
| Before vaccination |  |  |  |  |  |  |  |  |  |  |
| GMT | 18 |  | 14 | 0.66 |  | 12 | 0.17 |  | 15 | 0.65 |
| proportion ≥1:40 | 0.38 |  | 0.26 | 0.70 |  | 0.27 | 0.38 |  | 0.35 | 1.00 |
| 1 month after vaccination |  |  |  |  |  |  |  |  |  |  |
| GMT | 24 |  | 26 | 0.87 |  | 20 | 0.53 |  | 20 | 0.66 |
| proportion ≥1:40 | 0.49 |  | 0.46 | 1.00 |  | 0.47 | 1.00 |  | 0.48 | 1.00 |
| GMTR | 1.0 |  | 2.0 | 0.37 |  | 2.0 | 0.40 |  | 1.0 | 1.00 |

Footnote: P-values obtained by combined Chi-square test and Wald test where appropriate. Geometric mean titer (GMT); Geometric mean titer ratio (GMTR).
