# Supplementary material for: The Effect of Age and Recent Influenza Vaccination History on the Immunogenicity and Efficacy of 2009–10 Seasonal Trivalent Inactivated Influenza Vaccination in Children
Source: PLoS One. 2013 Mar 12;8(3):e59077. doi: 10.1371/journal.pone.0059077 (PMC3595209; doi:10.1371/journal.pone.0059077)
Supplement: Table S2 — Comparison of antibody titers before and 1 month after receipt of 2009–10 trivalent inactivated influenza vaccine (TIV) in children 9–17 years of age with regard to their vaccination history. (DOCX) [file pone.0059077.s004.docx]

Table S2. Comparison of antibody titers before and 1 month after receipt of 2009-10 trivalent inactivated influenza vaccine (TIV) in children 9-17 years of age with regard to their vaccination history.

|  | Reference |  | Comparison 1 | |  | Comparison 2 | |  | Comparison 3 | |
| --- | --- | --- | --- | --- | --- | --- | --- | --- | --- | --- |
|  | Not received TIV in 2007-08 or 2008-09 |  | Received TIV in 2007-08 only | P-value |  | Received TIV in 2008-09 only | P-value |  | Received TIV in 2007-08 & 2008-09 | P-value |
|  | (n=221) |  | (n=22) |  |  | (n=78) |  |  | (n=15) |  |
| Seasonal A(H1N1) |  |  |  |  |  |  |  |  |  |  |
| Before vaccination |  |  |  |  |  |  |  |  |  |  |
| GMT | 25 |  | 51 | 0.14 |  | 115 | <0.01 |  | 96 | <0.01 |
| proportion ≥1:40 | 0.48 |  | 0.59 | 0.90 |  | 0.82 | <0.01 |  | 0.82 | 0.06 |
| 1 month after vaccination |  |  |  |  |  |  |  |  |  |  |
| GMT | 511 |  | 228 | 0.03 |  | 330 | 0.04 |  | 307 | 0.02 |
| proportion ≥1:40 | 0.96 |  | 0.86 | 0.08 |  | 0.96 | 1.00 |  | 1.00 | 0.81 |
| GMTR | 20.0 |  | 4.0 | <0.01 |  | 3.0 | <0.01 |  | 3.0 | <0.01 |
|  |  |  |  |  |  |  |  |  |  |  |
| Seasonal A(H3N2) |  |  |  |  |  |  |  |  |  |  |
| Before vaccination |  |  |  |  |  |  |  |  |  |  |
| GMT | 23 |  | 41 | 0.09 |  | 74 | <0.01 |  | 143 | <0.01 |
| proportion ≥1:40 | 0.47 |  | 0.63 | 0.46 |  | 0.73 | 0.01 |  | 0.82 | 0.04 |
| 1 month after vaccination |  |  |  |  |  |  |  |  |  |  |
| GMT | 557 |  | 465 | 0.42 |  | 345 | 0.03 |  | 366 | 0.03 |
| proportion ≥1:40 | 0.95 |  | 1.00 | 0.62 |  | 0.98 | 0.74 |  | 1.00 | 0.80 |
| GMTR | 24.0 |  | 11.0 | 0.04 |  | 5.0 | <0.01 |  | 3.0 | <0.01 |
|  |  |  |  |  |  |  |  |  |  |  |
| Seasonal B/Brisbane |  |  |  |  |  |  |  |  |  |  |
| Before vaccination |  |  |  |  |  |  |  |  |  |  |
| GMT | 7 |  | 13 | 0.06 |  | 14 | <0.01 |  | 29 | <0.01 |
| proportion ≥1:40 | 0.09 |  | 0.32 | 0.08 |  | 0.31 | 0.01 |  | 0.55 | <0.01 |
| 1 month after vaccination |  |  |  |  |  |  |  |  |  |  |
| GMT | 90 |  | 78 | 0.66 |  | 71 | 0.46 |  | 142 | 0.10 |
| proportion ≥1:40 | 0.75 |  | 0.82 | 0.66 |  | 0.69 | 0.34 |  | 0.93 | 0.16 |
| GMTR | 13.0 |  | 6.0 | 0.04 |  | 5.0 | <0.01 |  | 5.0 | 0.02 |
|  |  |  |  |  |  |  |  |  |  |  |
| Pandemic A(H1N1) |  |  |  |  |  |  |  |  |  |  |
| Before vaccination |  |  |  |  |  |  |  |  |  |  |
| GMT | 21 |  | 25 | 0.61 |  | 16 | 0.35 |  | 27 | 0.55 |
| proportion ≥1:40 | 0.44 |  | 0.46 | 0.96 |  | 0.32 | 0.16 |  | 0.53 | 0.53 |
| 1 month after vaccination |  |  |  |  |  |  |  |  |  |  |
| GMT | 26 |  | 29 | 0.78 |  | 43 | 0.14 |  | 68 | 0.03 |
| proportion ≥1:40 | 0.50 |  | 0.50 | 1.00 |  | 0.56 | 0.73 |  | 0.74 | 0.13 |
| GMTR | 1.0 |  | 1.0 | 0.64 |  | 3.0 | 0.02 |  | 3.0 | 0.07 |

Footnote: P-values obtained by combined Chi-square test and Wald test where appropriate. Geometric mean titer (GMT); Geometric mean titer ratio (GMTR).
