# Supplementary material for: The Effect of Age and Recent Influenza Vaccination History on the Immunogenicity and Efficacy of 2009–10 Seasonal Trivalent Inactivated Influenza Vaccination in Children
Source: PLoS One. 2013 Mar 12;8(3):e59077. doi: 10.1371/journal.pone.0059077 (PMC3595209; doi:10.1371/journal.pone.0059077)
Supplement: Table S3 — Comparison of antibody titers before and 1 month after receipt of placebo in children 6–8 years of age with regard to their vaccination history. (DOCX) [file pone.0059077.s005.docx]

Table S3. Comparison of antibody titers before and 1 month after receipt of placebo in children 6-8 years of age with regard to their vaccination history.

|  | Reference |  | Comparison 1 | |  | Comparison 2 | |  | Comparison 3 | |
| --- | --- | --- | --- | --- | --- | --- | --- | --- | --- | --- |
|  | Not received any vaccine in 2007-08 or 2008-09 |  | Received vaccine in 2007-08 only | P-value |  | Received vaccine in 2008-09 only | P-value |  | Received vaccine in 2007-08 & 2008-09 | P-value |
|  | (n=56) |  | (n=5) |  |  | (n=45) |  |  | (n=14) |  |
| Seasonal A(H1N1) |  |  |  |  |  |  |  |  |  |  |
| Before vaccination |  |  |  |  |  |  |  |  |  |  |
| GMT | 19 |  | 52 | 0.26 |  | 24 | 0.59 |  | 47 | 0.13 |
| proportion ≥1:40 | 0.35 |  | 0.62 | 0.62 |  | 0.42 | 1.00 |  | 0.57 | 0.32 |
| 1 month after vaccination |  |  |  |  |  |  |  |  |  |  |
| GMT | 20 |  | 28 | 0.73 |  | 30 | 0.39 |  | 74 | 0.03 |
| proportion ≥1:40 | 0.37 |  | 0.43 | 1.00 |  | 0.45 | 0.95 |  | 0.65 | 0.15 |
| GMTR | 1.0 |  | 1.0 | 0.28 |  | 1.0 | 0.54 |  | 2.0 | 0.21 |
|  |  |  |  |  |  |  |  |  |  |  |
| Seasonal A(H3N2) |  |  |  |  |  |  |  |  |  |  |
| Before vaccination |  |  |  |  |  |  |  |  |  |  |
| GMT | 83 |  | 73 | 0.89 |  | 202 | 0.05 |  | 177 | 0.16 |
| proportion ≥1:40 | 0.71 |  | 0.62 | 0.87 |  | 0.81 | 0.56 |  | 0.86 | 0.50 |
| 1 month after vaccination |  |  |  |  |  |  |  |  |  |  |
| GMT | 88 |  | 39 | 0.46 |  | 170 | 0.15 |  | 178 | 0.19 |
| proportion ≥1:40 | 0.74 |  | 0.43 | 0.21 |  | 0.81 | 0.68 |  | 0.85 | 0.61 |
| GMTR | 1.0 |  | 1.0 | 0.29 |  | 1.0 | 0.17 |  | 1.0 | 0.90 |
|  |  |  |  |  |  |  |  |  |  |  |
| Seasonal B |  |  |  |  |  |  |  |  |  |  |
| Before vaccination |  |  |  |  |  |  |  |  |  |  |
| GMT | 8 |  | 5 | <0.01 |  | 7 | 0.48 |  | 12 | 0.45 |
| proportion ≥1:40 | 0.14 |  | 0.00 | 0.79 |  | 0.08 | 0.48 |  | 0.28 | 0.18 |
| 1 month after vaccination |  |  |  |  |  |  |  |  |  |  |
| GMT | 8 |  | 5 | 0.01 |  | 6 | 0.19 |  | 12 | 0.22 |
| proportion ≥1:40 | 0.11 |  | 0.00 | 0.87 |  | 0.04 | 0.30 |  | 0.35 | 0.01 |
| GMTR | 1.0 |  | 1.0 | 0.09 |  | 1.0 | 0.72 |  | 1.0 | 0.43 |
|  |  |  |  |  |  |  |  |  |  |  |
| Pandemic A(H1N1) |  |  |  |  |  |  |  |  |  |  |
| Before vaccination |  |  |  |  |  |  |  |  |  |  |
| GMT | 15 |  | 8 | 0.27 |  | 19 | 0.63 |  | 13 | 0.77 |
| proportion ≥1:40 | 0.36 |  | 0.19 | 0.72 |  | 0.42 | 0.58 |  | 0.28 | 0.74 |
| 1 month after vaccination |  |  |  |  |  |  |  |  |  |  |
| GMT | 21 |  | 14 | 0.58 |  | 25 | 0.62 |  | 16 | 0.64 |
| proportion ≥1:40 | 0.46 |  | 0.38 | 1.00 |  | 0.55 | 0.44 |  | 0.35 | 0.54 |
| GMTR | 1.0 |  | 2.0 | 0.64 |  | 1.0 | 0.99 |  | 1.0 | 0.72 |

Footnote: P-values obtained by combined Chi-square test and Wald test where appropriate. Geometric mean titer (GMT); Geometric mean titer ratio (GMTR).
