# Supplementary material for: The Effect of Age and Recent Influenza Vaccination History on the Immunogenicity and Efficacy of 2009–10 Seasonal Trivalent Inactivated Influenza Vaccination in Children
Source: PLoS One. 2013 Mar 12;8(3):e59077. doi: 10.1371/journal.pone.0059077 (PMC3595209; doi:10.1371/journal.pone.0059077)
Supplement: Table S4 — Comparison of antibody titers before and 1 month after receipt of placebo in children 9–17 years of age with regard to their vaccination history. (DOCX) [file pone.0059077.s006.docx]

Table S4. Comparison of antibody titers before and 1 month after receipt of placebo in children 9-17 years of age with regard to their vaccination history.

|  | Reference |  | Comparison 1 | |  | Comparison 2 | |  | Comparison 3 | |
| --- | --- | --- | --- | --- | --- | --- | --- | --- | --- | --- |
|  | Not received any vaccine in 2007-08 or 2008-09 |  | Received vaccine in 2007-08 only | P-value |  | Received vaccine in 2008-09 only | P-value |  | Received vaccine in 2007-08 & 2008-09 | P-value |
|  | (n=143) |  | (n=16) |  |  | (n=66) |  |  | (n=24) |  |
| **Seasonal A(H1N1)** |  |  |  |  |  |  |  |  |  |  |
| Before vaccination |  |  |  |  |  |  |  |  |  |  |
| GMT | 24 |  | 71 | <0.01 |  | 120 | <0.01 |  | 70 | <0.01 |
| proportion ≥1:40 | 0.47 |  | 0.82 | 0.07 |  | 0.78 | 0.03 |  | 0.75 | 0.10 |
| 1 month after vaccination |  |  |  |  |  |  |  |  |  |  |
| GMT | 28 |  | 69 | 0.02 |  | 168 | <0.01 |  | 84 | <0.01 |
| proportion ≥1:40 | 0.50 |  | 0.82 | 0.11 |  | 0.85 | 0.01 |  | 0.76 | 0.14 |
| GMTR | 1.0 |  | 1.0 | 0.24 |  | 1.0 | 0.51 |  | 1.0 | 0.92 |
|  |  |  |  |  |  |  |  |  |  |  |
| **Seasonal A(H3N2)** |  |  |  |  |  |  |  |  |  |  |
| Before vaccination |  |  |  |  |  |  |  |  |  |  |
| GMT | 28 |  | 48 | 0.19 |  | 85 | <0.01 |  | 160 | <0.01 |
| proportion ≥1:40 | 0.50 |  | 0.68 | 0.65 |  | 0.80 | 0.03 |  | 0.84 | 0.02 |
| 1 month after vaccination |  |  |  |  |  |  |  |  |  |  |
| GMT | 28 |  | 46 | 0.27 |  | 163 | <0.01 |  | 136 | <0.01 |
| proportion ≥1:40 | 0.48 |  | 0.62 | 0.97 |  | 0.89 | <0.01 |  | 0.84 | 0.01 |
| GMTR | 1.0 |  | 1.0 | 0.81 |  | 2.0 | 0.02 |  | 1.0 | 0.38 |
|  |  |  |  |  |  |  |  |  |  |  |
| **Seasonal B** |  |  |  |  |  |  |  |  |  |  |
| Before vaccination |  |  |  |  |  |  |  |  |  |  |
| GMT | 7 |  | 13 | 0.05 |  | 8 | 0.31 |  | 19 | <0.01 |
| proportion ≥1:40 | 0.06 |  | 0.31 | 0.08 |  | 0.11 | 0.93 |  | 0.46 | <0.01 |
| 1 month after vaccination |  |  |  |  |  |  |  |  |  |  |
| GMT | 7 |  | 10 | 0.30 |  | 9 | 0.53 |  | 20 | <0.01 |
| proportion ≥1:40 | 0.11 |  | 0.25 | 0.55 |  | 0.15 | 1.00 |  | 0.45 | <0.01 |
| GMTR | 1.0 |  | 1.0 | 0.04 |  | 1.0 | 0.94 |  | 1.0 | 0.77 |
|  |  |  |  |  |  |  |  |  |  |  |
| **Pandemic A(H1N1)** |  |  |  |  |  |  |  |  |  |  |
| Before vaccination |  |  |  |  |  |  |  |  |  |  |
| GMT | 19 |  | 22 | 0.72 |  | 16 | 0.54 |  | 15 | 0.60 |
| proportion ≥1:40 | 0.39 |  | 0.45 | 0.73 |  | 0.33 | 0.72 |  | 0.30 | 0.59 |
| 1 month after vaccination |  |  |  |  |  |  |  |  |  |  |
| GMT | 22 |  | 27 | 0.63 |  | 20 | 0.75 |  | 42 | 0.09 |
| proportion ≥1:40 | 0.45 |  | 0.57 | 0.58 |  | 0.42 | 0.69 |  | 0.63 | 0.16 |
| GMTR | 1.0 |  | 1.0 | 0.82 |  | 1.0 | 0.70 |  | 3.0 | 0.02 |

Footnote: P-values obtained by combined Chi-square test and Wald test where appropriate. Geometric mean titer (GMT); Geometric mean titer ratio (GMTR).
